# Influence of Different Percentages of Binders on the Physico-Mechanical Properties of *Rhizophora* spp. Particleboard as Natural-Based Tissue-Equivalent Phantom for Radiation Dosimetry Applications

**DOI:** 10.3390/polym13111868

**Published:** 2021-06-04

**Authors:** Siti Hajar Zuber, Nurul Ab. Aziz Hashikin, Mohd Fahmi Mohd Yusof, Mohd Zahri Abdul Aziz, Rokiah Hashim

**Affiliations:** 1 School of Physics, Universiti Sains Malaysia, Penang 11800, Malaysia; hajarzuber@student.usm.my; 2 School of Health Sciences, Universiti Sains Malaysia, Kelantan 16150, Malaysia; mfahmi@usm.my; 3 Advanced Medical and Dental Institute, Universiti Sains Malaysia, Penang 13200, Malaysia; mohdzahri@usm.my; 4 School of Industrial Technology, Universiti Sains Malaysia, Penang 11800, Malaysia; hrokiah@usm.my

**Keywords:** characterisation, particleboard, natural fiber composites, *Rhizophora* spp.

## Abstract

*Rhizophora* spp. particleboard with the incorporation of lignin and soy flour as binders were fabricated and the influence of different percentages of lignin and soy flour (0%, 6% and 12%) on the physico-mechanical properties of the particleboard were studied. The samples were characterised by Fourier transform infrared spectroscopy (FTIR), X-ray diffraction (XRD), scanning electron microscopy (SEM), energy dispersive X-ray (EDX), X-ray fluorescence (XRF) and internal bonding. The results stipulated that the addition of binders in the fabrication of the particleboard did not change the functional groups according to the FTIR spectrum. For XRD, addition of binders did not reveal any major transformation within the composites. SEM and EDX analyses for all percentages of binders added showed no apparent disparity; however, it is important to note that the incorporation of binders allows better bonding between the molecules. In XRF analysis, lower percentage of chlorine in the adhesive-bonded samples may be advantageous in maintaining the natural properties of the particleboard. In internal bonding, increased internal bond strength in samples with binders may indicate better structural integrity and physico-mechanical strength. In conclusion, the incorporation of lignin and soy flour as binders may potentially strengthen and fortify the particleboard, thus, can be a reliable phantom in radiation dosimetry applications.

## 1. Introduction

In medical physics, phantoms are used as a substitute for humans for various experiments and treatment verification purposes. Since ionising radiation is usually involved, irradiation of human subjects is not allowed, as radiation poses health risks for developing cancers in the future. Phantoms are materials comprising of elemental composition and density close to human tissue, and possess equivalent radiation properties (i.e., they generate similar interactions when irradiated). Acrylic (poly(methyl methacrylate)), a type of thermoplastic, is the most common, commercially available and widely used tissue-equivalent phantom material. Although it is biocompatible and is recyclable, it is not biodegradable and recycling this material usually leads to environmentally harmful by-products. Recent statistic shows that more than 380 million tonnes of plastic are produced every year, and due to their non-biodegradable properties, these plastic wastes may end up as pollutants and affect the natural environment and oceans [1].

Wood is an example of naturally occurring, bio-degradable composite materials that can be considered to replace acrylic as a phantom material. Wood is made up of fibrous chains of cellulose molecules in a matrix of organic polymer lignin. Cellulose fibres from wood are part of natural fibres, and these fibres are among the most abundant and renewable resources, eco-friendly and low cost. Composite materials are often used in constructions, especially in building reinforced structures, bridges and composites products such as particleboard, fibre-reinforced polymer or fiberglass [2,3]. Natural fiber composites are also readily used for interior finishes such as, interior door panels and floor mats [4]. Composite materials are advantageous in terms of their flexibility in design, light weight, physical strength, durability and good resistance toward corrosion [5,6]. Particleboard is a wood-based composite panel product consisting of cellulosic particles of various sizes that may be bonded together with any binders, under the influence of heat and pressure. *Rhizophora* spp. was investigated and show potential as durable particleboard in the construction of furniture, interior applications and as tools in medical applications [7,8,9,10]. In radiation dosimetry applications, *Rhizophora* spp. has been studied for their potential as a phantom material and several studies have proved their properties as a tissue-equivalent material [10,11,12]. The composition and electrical properties of the tissue-mimicking phantom material are also important for better modelling of the phantom, adhering to the body structure represented by the phantom [13]. In order to develop composite phantom materials with tailored properties, one should be able to predict a property of a composite based on the properties of its constituents [14].

Adhesives, also known as binders, may be found naturally or produced synthetically [15]. Wood binder is used in most wood products including particleboards, boards, plywood and many other applications. Over the years, the development of binders in the wood industry shows significant changes, with higher production numbers, and with the emergence of synthetic binders, the range of binder formulations exploded. Commercial synthetic binders are commonly used in the wood industry, which include urea formaldehyde (UF), phenol formaldehyde (PF) and phenol resorcinol formaldehyde (PRF) [16,17]. This is due to their properties, which include excellent water resistance, and they provide better performance than a natural binder [18].

Despite popular use of synthetic resins in the wood industry, several harmful environmental effects were discovered [19,20]. In 2008, the Environmental Protection Agency (EPA) declared formaldehyde a carcinogenic material which brings about environmental and health concerns [20,21,22,23]. Among the side effects of formaldehyde towards humans are inflammation of the eyes, nose, throat, asthma-like respiratory allergy, asthma attack with shortness of breath, wheezing and coughing. In the environmental aspect, formaldehyde decomposes in air and forms formic acid and carbon monoxide. Animals that are affected by formaldehyde may experience chronic effects which include, shortened lifespan, reproductive problems and lower fertility. As a result, several attempts have been made to improve environmentally friendly binders in an effort to substitute formaldehyde as a binder in the wood industry.

Soy protein is one of the natural resources to produce natural wood adhesive [19]. Soy products are preferred as binders due to several factors which include, availability, being economical and that presence of carbohydrate in soy products serve as an inert dilute, which is an excellent addition in adhesive materials. They are also safe and perform well as a dominant bonding portion [24]. Other than that, soy protein can withstand hot or cold conditions during the fabrication process, and more efforts have been done to study soy protein as a binder in order to improve the wood bond strength [24,25,26,27,28,29]. Soy protein is also acknowledged for its good adhesive properties, due to its structures and several modifications. The structure of soy protein can be modified to adhere to the requirement, which may improve its properties. Hydrogen bond presented in the soy protein structure prevents it from interacting with the cellulosic structure of the wood; however, this disadvantage can be overcome by several modifications. The molecular structures of the soy protein can be modified by using physical, chemical or enzymatic means. The modifications result in the breaking down of hydrogen bond, allowing the soy protein structure to be able to react with the wood material with increased adhesion properties.

Lignin is part of the components that hold the plant fibers together and this property roused interests for it to be utilized as a suitable wood binder, which is low-cost, non-toxic, easily available, renewable and environmental-friendly. Lignin consists of approximately 40% of wood’s mass and is among the most abundant natural polymers on earth, accounting for 12% to 33% of lignocellulosic biomass [30,31]. Lignin is often studied for the production of carbon fibers, thermoplastics and binders [32,33,34,35]. It has been studied as a binder for hundreds of years but the development of a binding system, mainly from lignin, has yet to be carried out [36]. Kraft lignin and lignosulfonates from hardwoods and agricultural residues are among the lignin products that show promising potentials as good wood binders, and more efforts have been done to make binder blends based on these two products [37,38,39,40].

There are many studies devoted to the characterisation of natural based composites and polymer as a phantom in radiation dosimetry applications; however, the study of the effect of different percentages of binders towards the characterisation outcomes and physico-mechanical properties are yet to be discussed. In this study, different percentages of lignin and soy flour were formulated as binders in the fabrication of *Rhizophora* spp. particleboard, to study their effects towards different analyses: i.e. Fourier transform infrared (FTIR), X-ray diffraction (XRD), scanning electron microscopy (SEM), energy dispersive X-ray (EDX) and X-ray fluorescence (XRF). Internal bonding analysis was also performed.

## 2. Materials and Methods

### 2.1. Sample Preparation of Rhizophora spp. Particleboard Bonded with Lignin and Soy Flour

*Rhizophora* spp. raw wood trunks were obtained from a coal factory in Perak and underwent several steps, which include drying, debarking, grinding and sieving, to prepare wood particles at approximately 0 µm to 103 µm particle sizes. Lignin (Sigma Aldrich, Merck, QRec, Petaling Jaya, Malaysia) and soy flour (Sigma Aldrich, Merck, QRec, Petaling Jaya, Malaysia) were utilised as binders for the fabrication of the particleboard. Different percentages of binders, i.e. 0%, 6% (3:1 soy flour to lignin) and 12% (3:1 soy flour to lignin) binders, were pre-determined to analyse their effects towards the characterisation and physico-mechanical properties. Bonded particleboards were fabricated at a target density of 1.0 g.cm^−3^, while maintaining the *Rhizophora* spp. moisture content at approximately 1.39% L to 6.47% L. Moisture content of the sample is best being below 10% L to maintain its relative humidity, and may also be affected by type, size and geometry of the sample [15]. The fabrication process involved hot pressing by using a hot press machine at approximately 200 °C, with a pressure of 20 MPa for approximately 17 min to 20 min.

### 2.2. Fourier Transform Infrared (FTIR) Analysis

Analysis of the structure of the molecules and functional groups present in the samples were determined using an FTIR spectroscopy, to investigate the chemical bonding with the addition of soy flour and lignin as binders. The samples were ground into approximately 1.0 mg of powder, mixed with approximately <100 mg of potassium bromide (KBr), and was then compressed to form 1.0 mm thickness of pellet. In this analysis, FTIR spectrophotometer (IRPrestige21, Shimadzu, Japan) at the School of Industrial Technology, Universiti Sains Malaysia was utilized.

### 2.3. X-ray Diffraction (XRD) Analysis

XRD analysis was carried out using Bruker’s D8 Advance X-Ray Diffractometer at the Center of Global Archeological Research, Universiti Sains Malaysia, to estimate the degree of crystallinity of the fabricated samples. On the sample holder, the powdered samples were hydraulically pressed into 25 mm diameter circular discs. The samples were radiated at energy of 40 kVp. The source used was Cu-K_α1_, with wavelength λ of 1.54060 Å, scanning range of approximately 5° 2θ to 70° 2θ and scanning speed of 0.02° 2θ/s.

### 2.4. Analysis of Scanning Electron Microscopy (SEM) and Energy Dispersive X-ray (EDX)

The microstructures and elemental compositions of the bonded *Rhizophora* spp. samples were obtained and studied using a SEM (FEI Quanta FEG-650, Eindhoven, The Netherlands) and EDX at the Centre for Global Archeological Research, Earth Material Characterisation Laboratory, Universiti Sains Malaysia. The samples with the measurement of (5.0 × 5.0 × 0.5) cm^3^ were taped to a specimen holder with two-sided adhesive tape, and coated with gold by a sputter coater (Quorum Q150T S, Quantum Design GmbH, Darmstadt, Germany). The images were taken at a magnification of 4000×. For EDX analysis, the elements were preset to focus only on carbon, nitrogen and oxygen.

### 2.5. X-ray Fluorescence (XRF) Analysis Using Omnian Analysis Software

The samples were analysed using a 4 kW wavelength dispersive XRF machine (Axios Max, Panalytical, Almelo, Holland, The Netherlands). This machine has a maximum voltage of up to 60 kV and 160 microamps (µA), and is equipped with several crystals such as, PE002, LiF200, PX1 and Ge 111. A representative portion of each sample was ground into 50 µm grain size using a motorised grinding machine, and was further ground manually to a finer grain size of 20 µm, suitable for XRF analysis. The specimen for the XRF analysis was made by igniting approximately 0.5 g of sample and 5.0 g of spectroflux, at approximately 1100 °C (for a duration of 20 min), before it was casted into a glass disc with 32 mm in diameter. The specimen was analysed for 10 major elements using a fully automated XRF spectrometer (Axios Max, Panalytical, Almelo, Holland, The Netherlands), with a standard elemental setup. The calibration technique was employed. The 10 element curves were constructed using 30 high quality international standard reference materials, comparable in composition to the unknown samples. For minor and trace elements, each sample was formed into pressed-powder pellet, using approximately 1 g of sample baked with approximately 6.0 g of Boric acid (in a 32 mm diameter round shaped disc, with the sample placed at the center, and Boric acid as a binder around it). The samples were pressed with a hydraulic press machine at 15 tonnes for 2 min. The XRF analysis was done by scanning for the presence of elemental peaks using the Omnian software. The weight percentages of the compound presented in the samples were recorded in the results upon the completion of the analysis.

### 2.6. Internal Bonding Analysis

Internal bond analysis was performed for the fabricated *Rhizophora* spp. bonded with different percentages of binders [8]. Metal blocks with the dimension of approximately (5.0 × 5.0) cm^2^ were utilised in this analysis, and a hot melt glue was used to bind the samples to the metal blocks. Internal bond strength was determined by using a mechanical testing machine (Model UTM-5582; Instron, Norwood, MA, USA) with a load capacity of approximately 1000 kg, adapted from a previous study by Zuber et al. [8]. 

## 3. Results and Discussion

### 3.1. Evaluation of Functional Group using FTIR Analysis

**Figure 1:**
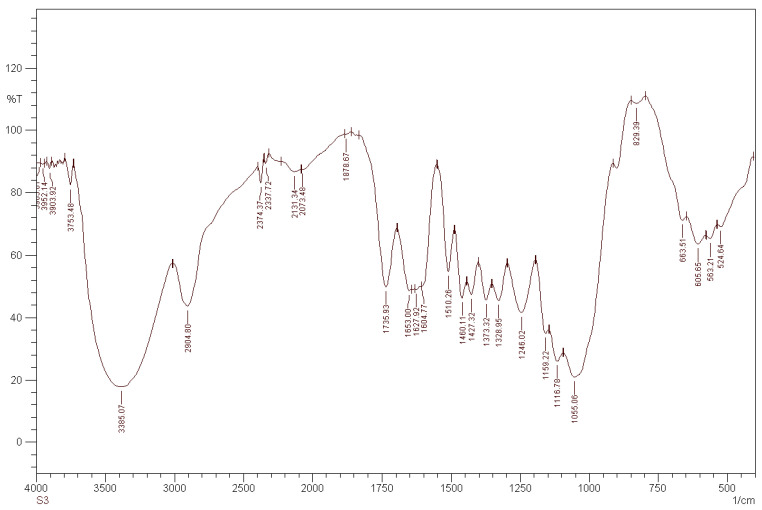
Transmittance spectrum of *Rhizophora* spp. particleboard bonded with 0% binder.

**Figure 2:**
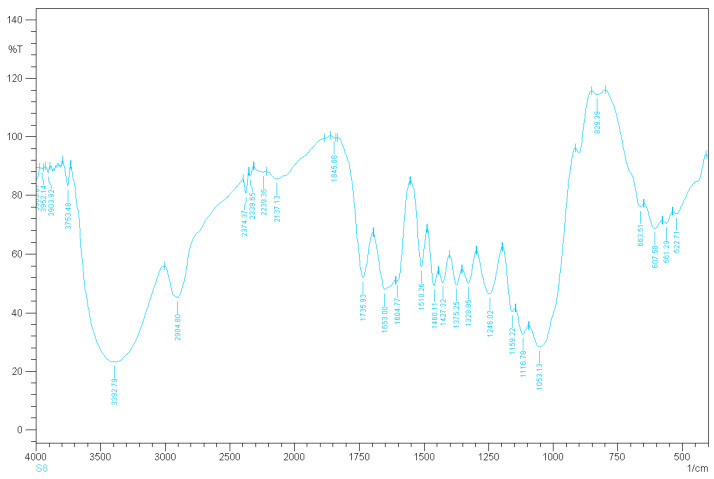
Transmittance spectrum of *Rhizophora* spp. particleboard bonded with 6% binders.

**Figure 3:**
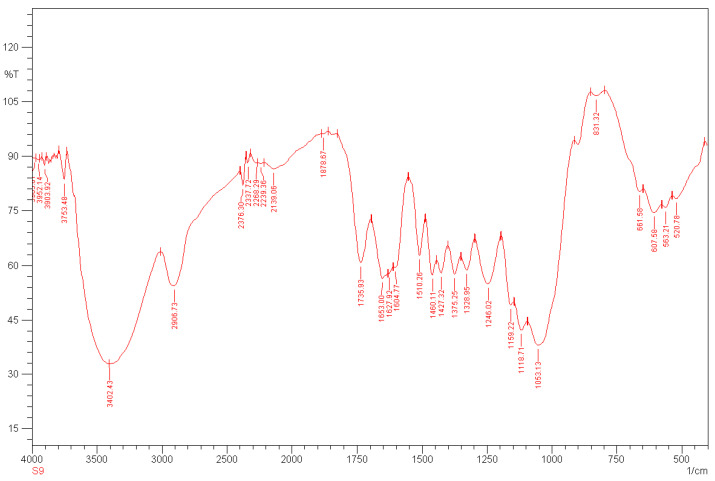
Transmittance spectrum of *Rhizophora* spp. particleboard bonded with 12% binders. Reprinted with permission from ref. [7].

The analyses for the *Rhizophora* spp. particleboard bonded with different percentages of binders were recorded between wavenumbers of 4000 cm^−1^ and 400 cm^−1^, with a resolution of 4 cm^−1^. Different relative transmittance values were determined by FTIR spectral analysis, as shown in Figure 1, Figure 2 and Figure 3. Based on the overview of the FTIR values for cellulose, hemicellulose, saccharides and lignin, the broad peak at 3385.07 cm^−1^ in Figure 1 may represent cellulose and hemicellulose related to O-H stretching, in relation to hydrogen bond of hydroxyl groups [41,42]. The peak at 2904.80 cm^−1^ may indicate cellulose in relation to C-H stretching, whereas the peak at 1735.93 cm^−1^ may represent the softwood or hardwood with C=O stretching [41]. All the samples displayed almost the same spectra, as indicated by all the peaks presented in the Figure 1, Figure 2 and Figure 3. Compared to the spectrum of binderless *Rhizophora* spp. sample, the spectra of samples incorporated with lignin and soy flour as binders showed a small increase in the peak intensity, as shown in peak 3402.43 cm^−1^ and 2809.80 cm^−1^. However, no new peak appeared in the spectrum of *Rhizophora* spp. samples bonded with the binders, indicating that the introduction of lignin and soy flour did not change the functional groups according to the FTIR spectrum.

### 3.2. Evaluation of XRD Spectrum

**Figure 4:**
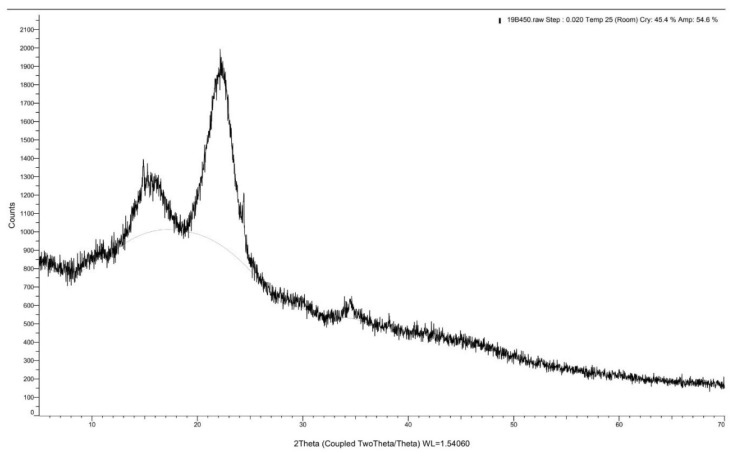
X-ray diffraction spectrum for *Rhizophora* spp. particleboard bonded with 0% binder.

**Figure 5:**
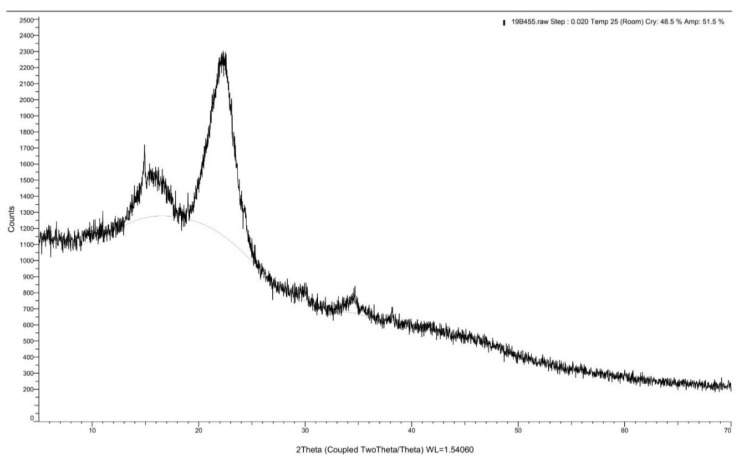
X-ray diffraction spectrum for *Rhizophora* spp. particleboard bonded with 6% binders.

**Figure 6:**
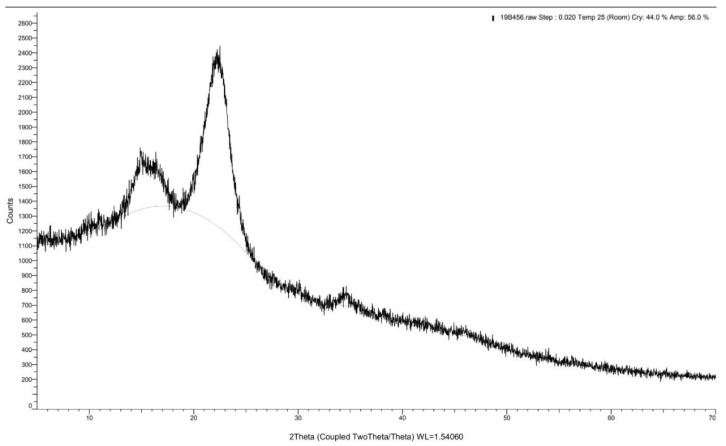
X-ray diffraction spectrum for *Rhizophora* spp. particleboard bonded with 12% binders Reprinted with permission from ref. [7].

XRD spectrums of the *Rhizophora* spp. bonded with different percentages of binders are depicted in Figure 4, Figure 5 and Figure 6. A peak intensity at around 2θ = 22° was observed in the diffractogram of the sample bonded with 0% binders, whereas for samples with 6% and 12% binders, the peak intensity can be seen at 2θ = 22.20°, which corresponds to the crystalline properties of the composites [43]. The addition of lignin and soy flour led to a small increment of peak intensity at approximately 2θ = 22.20°, suggesting that the introduction of binders may indicate the changes in the structural order of the molecules, which may also influence the crystalline structure of the composites [44]. This may be due to the forces from molecular chain entanglements between the *Rhizophora* spp. wood particles and the binders, as they diffuse across the joint interface. Cross-linking may occur during the hot pressing, while interfacial diffusion during bonding is enhanced by chain scission [45]. However, no apparent disparity can be observed in all the spectrums, indicating that no major structural transformation can be clearly stipulated in this analysis. To conclude, incorporation of lignin and soy flour in the fabrication of *Rhizophora* spp. particleboard did not reveal apparent crystalline and amorphous transformations within the composites.

### 3.3. Evaluation of SEM Analysis

**Figure 7:**
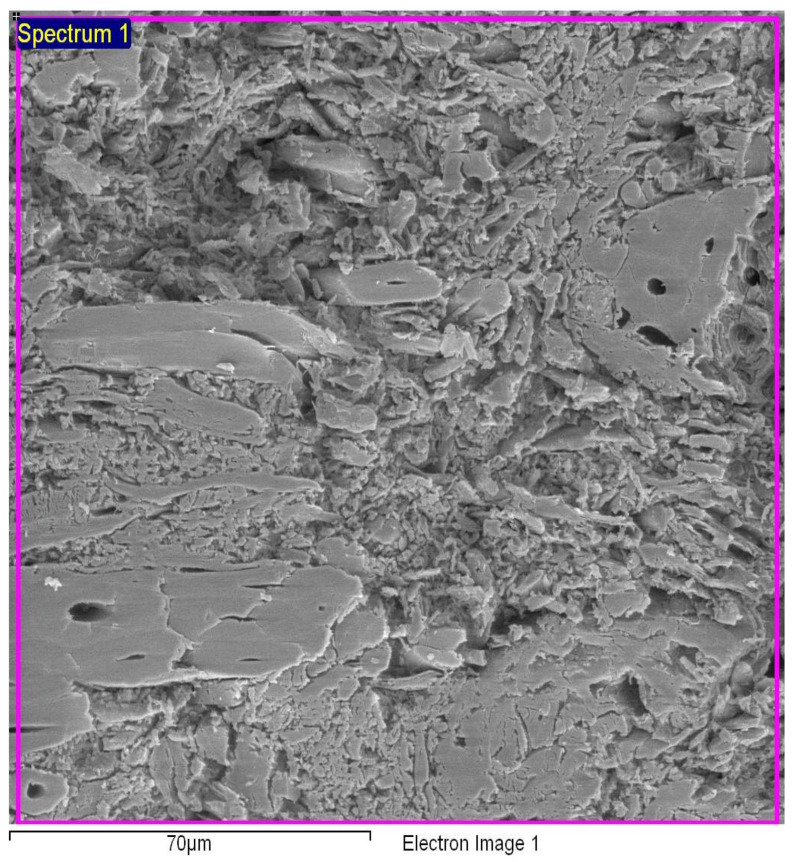
Micrograph of *Rhizophora* spp. particleboard bonded with 0% binder.

**Figure 8:**
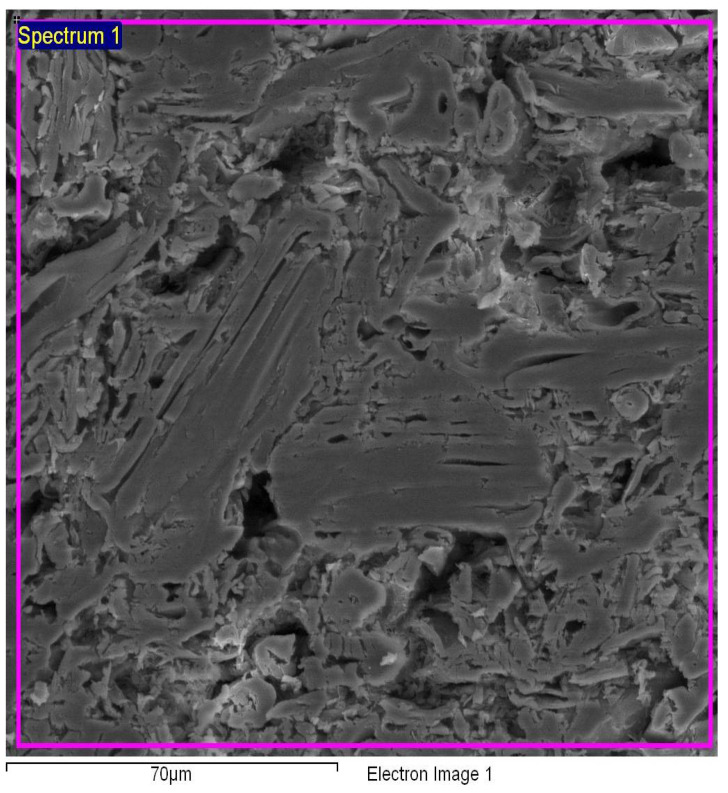
Micrograph of *Rhizophora* spp. particleboard bonded with 6% binders.

**Figure 9:**
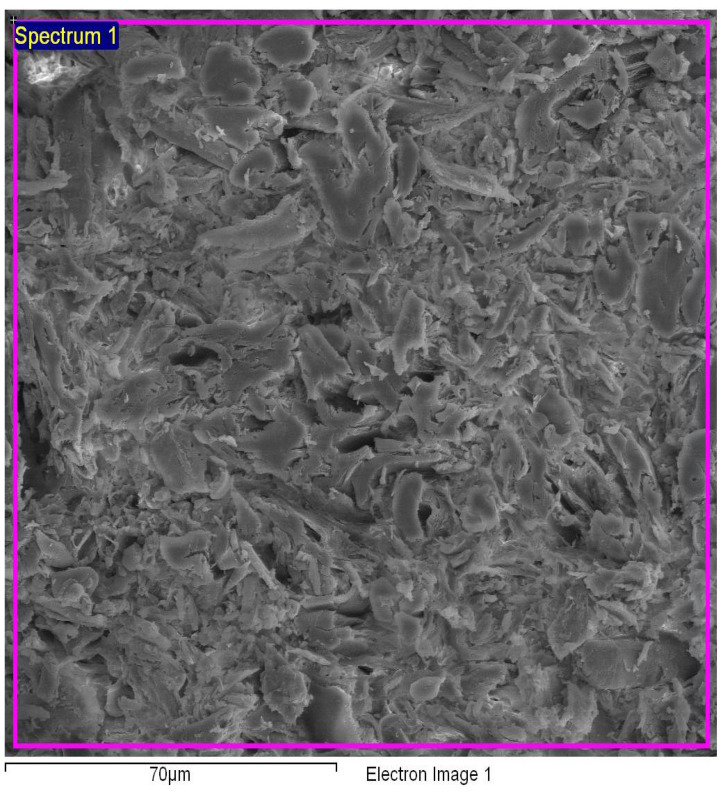
Micrograph of *Rhizophora* spp. particleboard bonded with 12% binders.

The micrographs of the particleboards with different percentages of binders are depicted in Figure 7, Figure 8 and Figure 9. For the sample with 6% binders, the void spaces between the molecules were reduced when compared to the binderless sample, demonstrating a much smoother appearance which can be attributed to the hot pressing that allows the auto-condensation process between the wood particles and the binders, binding them together [7]. For the *Rhizophora* spp. sample with 12% binders as shown in Figure 9, the appearance of the molecules revealed a less smooth appearance, which may be due to insufficient bonding between the wood particles and the binders, causing an increase in lumen and gaps. Increased lignin content can affect the composites’ compactness as cross-linking of lignin with the cell wall components may occur, which in return, reduce cellulose and hemicellulose accessibility to microbial enzymes, resulting in lower biomass digestibility [46]. Specks of bright appearance in all the figures may be due to the charging effect of electron or ion irradiations, which usually happens for a non-conductive specimen being examined [47]. The interactions between *Rhizophora* spp. and the binders were also influenced by the intermolecular forces that mediate the interaction, and the forces of attraction and repulsion between the molecules [25,48,49,50]. These attractive forces may allow better adhesion between the binders in their molecular forms, together with the raw wood particles. The natural properties of the raw wood itself are an advantage due to its cell cavities, which may allow the binders to infiltrates and provide better bonding with the help of thermal pressing. Data adapted from Zuber et al. revealed the thermogravimetric analysis (TGA) results of *Rhizophora* spp., soy flour and lignin. The mass degradation of *Rhizophora* spp., soy flour and lignin occurred at 303.35 °C, 236.11 °C and 279.64 °C, respectively, where the particles started to decompose at these temperatures. The mass degradation temperature should not be exceeded in the compression process of the particleboard, in order to improve the efficiency between the fibre chain of the particles and the binders [7]. The process of auto-condensation during the hot pressing had bound the molecules of the binders together with the *Rhizophora* spp. fibre; however, depending on the formulation of the composites, the microscopic appearances of the samples may slightly differ. In this study, the distribution of the *Rhizophora* spp. particles and binders was influenced by shrinking and compounding of the composites to a specified thickness by hot pressing at approximately 200 °C, leading to good interfacial bonding. Although there is no apparent disparity between the micrographs shown, it is important to note that incorporation of lignin and soy flour in the fabrication of the particleboard allows better bonding between the molecules, which will further improve the physical and mechanical strength of the particleboard [8].

### 3.4. Evaluation of EDX Analysis

**Figure 10:**
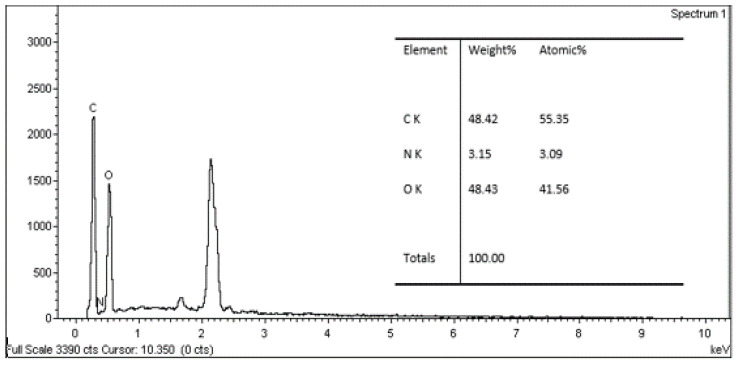
Energy dispersive X-ray spectrum for *Rhizophora* spp. particleboard bonded with 0% binders.

**Figure 11:**
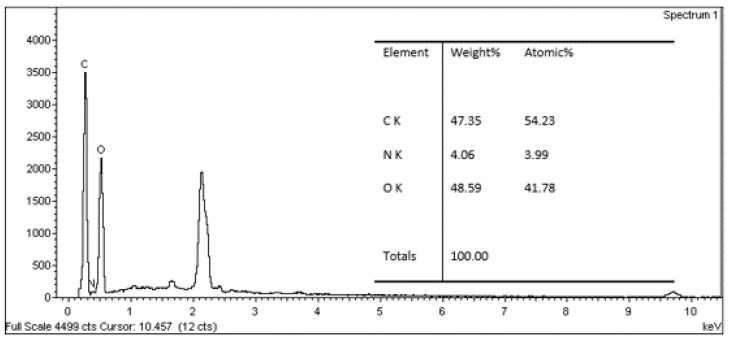
Energy dispersive X-ray spectrum for *Rhizophora* spp. particleboard bonded with 6% binders.

**Figure 12:**
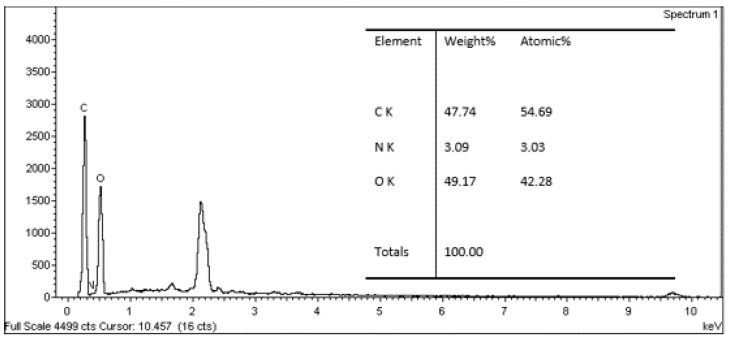
Energy dispersive X-ray spectrum for *Rhizophora* spp. particleboard bonded with 12% binders.

Figure 10, Figure 11 and Figure 12 show EDX spectrums of the *Rhizophora* spp. particleboard at 0%, 6% and 12% binders. All the EDX spectrums of *Rhizophora* spp. particleboard bonded with different percentages of binders demonstrated visible carbon and oxygen signals, which confirmed the discernible presence of the carbon and oxygen in the composites [51,52]. Another peak in the spectrums may represent other elements such as gold, as the samples were coated with gold for better conductivity. Based on the figures, all spectrums display no discernible disparity; thus, the incorporation of binders in the fabrication of *Rhizophora* spp. particleboard did not greatly affect the percentages of elements presented in the composites.

### 3.5. Evaluation of the XRF Analysis

The XRF method is the best way to identify major and trace elements within the natural composition of the particleboard. The XRF analysis of major elements is recorded in Table 1. Calcium oxide showed the highest weight percentage in all samples in the range of 40.319% to 52.744%. For *Rhizophora* spp. samples with the addition of lignin and soy flour, the dry weight of potassium oxide recorded the second highest concentration in the range of 14.883% to 18.399%, while chlorine percentage is between 11.623% to 12.947%. However, for the binderless particleboard (0% soy flour and lignin), chlorine recorded the second highest percentage after calcium oxide at 17.705%, followed by sodium oxide at 6.355%. The high percentage of chlorine in the wood-based sample may be due to combustion performed in a laboratory scale spectrometer. Nevertheless, the lower percentage of chlorine in the bonded samples may be advantageous to the overall particleboard outcomes, especially in maintaining the natural properties of the particleboard.

### 3.6. Internal Bond Analysis

**Figure 13:**
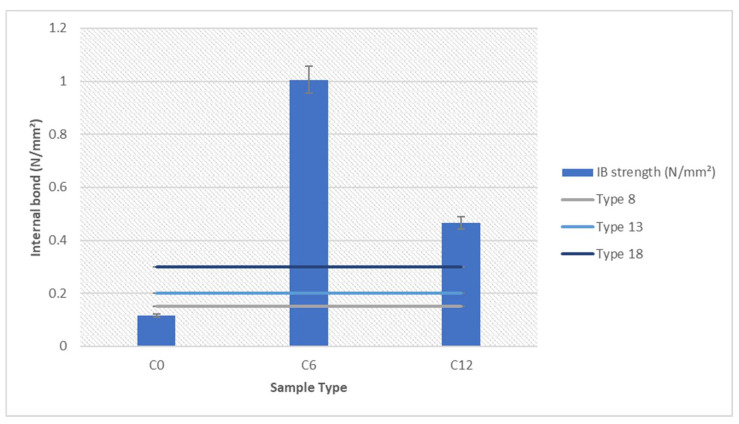
Internal bonding of soy-lignin bonded *Rhizophora* spp. particleboards at different percentages. C = 0 µm to 103 µm particle sizes; 0 = 0% soy flour and lignin, 6 = 4.5% soy flour and 1.5% lignin, 12 = 9% soy flour and 3% lignin.

The analysis of internal bonding was performed and the result is shown in Figure 13 [8].

The minimum requirement according to JIS A-5908 includes classification of Type 8 (0.15 N.mm^−2^), Type 13 (0.2 N.mm−^2^) and Type 18 (0.3 N.mm^−2^) [53]. The *Rhizophora* spp. sample with binders satisfy all three classifications by JIS, whereas the sample with 0% binder did not satisfy any of the requirements. Increased internal bonding strength displayed by samples with binders indicates improved structural stability and durability of the composites [44]. Hot pressing allows the high temperature to be imparted on the formulation of *Rhizophora* spp. with binders, which may create strong bonding with reduced lumen voids between the particles in the composites. The strength of the particleboard may be achieved due to the vessel element and parenchymatous cell of the *Rhizophora* spp. that are closely attached under high pressure condition. In this study, internal bond represents the mechanical properties of the composites, and based on the result, the addition of lignin and soy flour increased the internal bond strength, which may improve its mechanical strength and structural integrity.

## 4. Conclusions

Evidence from the FTIR, XRD, SEM, EDX, XRF and internal bonding revealed the potential use of lignin and soy flour as binders in the fabrication of *Rhizophora* spp. particleboard, as a phantom material in radiation dosimetry applications. Different percentages of binders used in the fabrication of the particleboard did not greatly affect the properties of the particleboard as a phantom material; however, lower percentages of chlorine and increased internal bonding strength in the sample with binders may be advantageous in maintaining the natural properties of the particleboard, and improve the mechanical strength of the samples, respectively.

## Figures and Tables

**Table 1:** X-ray fluorescence analysis of major and trace element in weight percentages using Omnian method with pressed powder pellet.

Samples	Calcium Oxide (CaO)	Potassium Oxide (K_2_O)	Chlorine (Cl)	Sodium Oxide (Na_2_O)	Others
0% binder	52.744	5.241	17.705	6.355	17.955
6% binders	42.740	14.883	12.947	5.036	24.394
12% binders	40.319	18.399	11.623	4.708	24.951

## Data Availability

The data presented in this study are available on request from the corresponding author.
